# Extended Pharmacist Assessment of Medication Safety for Nursing Home Residents—A Cross-Sectional and Prospective Study

**DOI:** 10.3390/jcm11216602

**Published:** 2022-11-07

**Authors:** Christian Führling, Renke Maas

**Affiliations:** 1 Institute of Experimental and Clinical Pharmacology and Toxicology, Friedrich-Alexander-Universität Erlangen-Nürnberg, 91054 Erlangen, Germany; 2 Dr. Führling’s Apotheken, Mommsenstraße 22, 90491 Nürnberg, Germany

**Keywords:** medication safety, nursing home residents, medication review, medication related problem, medication error

## Abstract

In the routine pharmacist’s medication review in ambulatory care and nursing homes in Germany, clinical diagnoses are often insufficiently considered as they are frequently not accessible to pharmacists and their electronic support tools. This may leave a significant proportion of medication-related problems (MRP) undetected and unresolved. Moreover, limited and incomplete data may promote spurious alerts of low clinical relevance. In order to assess the impact of improved data availability, we conducted a study (German Clinical Trials Register DRKS00025346) to evaluate the impact of an extended pharmacist’s medication review, made possible by diagnosis data being routinely available to the pharmacist. At six nursing homes in the Nuremberg metropolitan area, 338 patients treated by 32 physicians were enrolled. A pharmacist’s medication review, considering only the medication data, identified 114 potential MRPs, and additional consideration of diagnoses further identified 69 potential MRPs. The physicians adapted the therapy in response to 69.9% of alerts. The observed gain in MRP identified indicates that efforts should be intensified to facilitate and improve consideration of drug–diagnosis-related MRP by improving data sharing and communication between pharmacists and physicians caring for nursing home residents.

## 1. Introduction

Residents of inpatient care facilities for the elderly represent a continuously growing population. Frailty, multimorbidity, and polymedication make this group especially prone and sensitive to medication-related problems [1]. Due to the increased prevalence of chronic diseases, the number of prescribed drugs and self-medications usually increases with age [2]. Drug safety in this population is characterized by age-associated changes in pharmacokinetics and pharmacodynamics, an increased risk for drug–drug interactions as well as comorbidities, which all increase the susceptibility to side effects [3]. Applying multiple guidelines in patients with multimorbidity can result in dangerous or contraindicated drug–drug and drug–disease interactions [4]. Moreover, the dosages of many drugs have to be modified to account for age or age-related comorbidities, such as reduced renal function [5]. Therefore, the recognition of medication-related problems (MRP) and the minimization of avoidable medication errors are of special importance in elderly patients. Several lists and criteria to identify potentially inadequate medication (PIM) in the elderly have been proposed, yet the prevalence of PIM in the elderly remains high [6]. 

If all prescriptions that have been prescribed to a patient by different doctors, and possibly also over-the-counter (“OTC”) drugs, are dispensed and recorded by a single pharmacy, the completeness of the information is improved and the chance of recognizing, at least, medication problems such as drug interactions, increases [6,7]. Investigations in emergency patients have shown that with the usual focus on the easy to (electronically) detect drug–drug interactions, a significant proportion of medication errors could possibly go undetected, as these often result from contraindicated “drug–diagnosis” combinations [8].

Drug therapy safety is undoubtedly an interdisciplinary task. This means that in their respective settings, all professional groups involved (i.e., physicians, pharmacists, nurses, medical assistants, and other healthcare professionals working with patients) must define their respective roles and responsibilities [9]. 

With healthcare systems becoming more and more digitalized, patients may expect that their physicians and pharmacists have access to all data relevant for medication safety (such as complete medication data, diagnoses, and laboratory values) and use comprehensive electronic medication safety checks when prescribing or dispensing a new medication. In clinical reality, this often is not yet the case [10].

Lack of information either about the patient, diagnoses and concurrent medications, or about the prescribed drug, its correct dosing, contraindications, and drug interactions often lead to preventable adverse drug events [11,12]. Information on medication and diagnoses relevant for safe prescriptions is often distributed across several health care providers, with no one having the complete overview of all drugs and diagnoses [13]. This carries a high risk of medication errors. Even the paper-based nationwide medication plan introduced in 2016 in Germany has hardly been able to change anything up to now, since its topicality, completeness, and suitability for the detection of medication problems in everyday life still has considerable limitations [7]. 

While it can be expected that additional consideration of diagnoses increases the number of MRP identified, unintended consequences such as over-alerting and a decline in acceptance may have to be considered as well. In contrast to simple drug–drug interaction alerts, MRP alerts related to clinical conditions may be more prone to false or clinically irrelevant alerts because it requires more complex clinical judgements. In addition, some medical information that is required for the correct interpretation regarding whether or not the application really constitutes a contraindication may not have been documented. For example, the diagnosis “asthma” may have been documented, but not its clinical severity, which may make a difference in judgement if a drug such as a beta-blocker is absolutely contraindicated or may be used with caution. Here, data regarding which warnings by the pharmacist lead to adaptation of the therapy and which do not, are of great practical interest, to reduce “false alarms”, but also to identify gaps in communication and information. 

The author’s pharmacies participated in a local effort to overcome these obstacles by improving the availability of medical data to the pharmacists to allow extended medication reviews and by establishing a simple standard of communication between pharmacists and physicians caring for nursing home patients. This setting provided the opportunity to assess the MRP detected and addressed by a conventional pharmacist review focused on the medication schedule, as compared to a pharmacist’s review, which also allowed consideration of key clinical diagnoses.

## 2. Materials and Methods

### 2.1. Setting and Definition of Routine Care

For the period from June 2021 to April 2022, the prospective part of this study was carried out in six nursing homes in the Nuremberg metropolitan area. All patients inscribed in a routine pharmaceutical care program, which permitted shared access of the pharmacists and the physicians to a patient’s key medical data, stored in the joint documentation of physicians and pharmacists in the nursing home, were eligible for this study. As part of this routine care arrangement, the pharmacist performs a standard medication review each time a new prescription is filled and communicates any MRP to the prescribing physician—who can be a general doctor or a specialist. This routine review is primarily confined to the assessment of the available medication data (including messages automatically generated by the standard pharmacy software), i.e., the assessment of simple (drug–drug) interactions, double medication, and obvious documentation problems such as missing dosing schedule, timely unclear intake, unclear intake breaks, or dosing errors irrespective of clinical indications of a drug. 

To avoid over-alerting in the routine care setting, as well as in the advanced care setting described further below, the software was set to flag only critical alerts. These primarily included warnings usually mandating a therapeutic consequence, such as “adjustment of medication possibly required” or higher (“contraindicated as a precaution” or “probably contraindicated”). Moreover, only warnings considered truly clinically relevant were forwarded by the pharmacist.

Formally, even warnings flagged as “contraindicated” were only considered and communicated as “potentially” contraindicated because there may have been (and sometimes was) a clinical reason justifying the use, which was not disclosed in the information available to the pharmacist performing the review.

For technical and legal reasons, fax was used for communication of medication alerts to physicians. If there was no response, a second fax was sent. If the physician still did not respond to the second fax either, prescriptions unchanged after the third report (third fax) to the doctor were documented as “tolerated”. Additional provisions were made to inform the patients or their caretakers directly in case of very urgent problems, but this condition was not met in the study period. 

### 2.2. Study

The study was approved (No. 329_20 B) by the state-accredited ethics board of the Friedrich-Alexander-Universität Erlangen-Nürnberg. Written informed consent was provided by the study participants or their legal representatives. The study also was registered at the German Clinical Trials Register (DRKS00025346).

### 2.3. Inclusion and Exclusion Criteria

Inclusion and exclusion of the patients were defined by the respective presence and absence of the following criteria: participation in the routine pharmaceutical care program, age > 65 years, the regular intake of at least five different drugs for the index prescription (*n*) and at least one previous prescription and informed consent.

### 2.4. Study Specific Measures

In patients enrolled in this study, the pharmaceutical review was extended to include possible medication problems related to the diagnoses documented in the local patient record as well. This allowed to take diagnosis-related contraindications into account, which, according to the manufacturer´s technical information, represent a contraindication for the administration of the respective drug involved, and which are not detected by the standard pharmacy software. The special software Scholz database was used as reference for this analysis—supplemented by search for the respective specialist information—which can support the pharmacist with extended functionality during analysis. The standard pharmacy software of the ABDA has been expanded to include the Scholz database for the additional assessment of drug–diagnosis interactions. This database is made available by the German Pharmacist´s Association and CE marked and certified according to ISO 9001: 2015 by the certification of TÜV SÜD and GKV/AVWG and was used within its label.

The intake of five or more active substances was defined as polypharmacy, a glomerular filtration rate (GFR) of less than 30 mL/min was defined as “impaired renal function or renal failure” of relevance for the majority of contraindications related to renal function.

### 2.5. Data Analysis

#### Anonymization of Patient Data

For analysis, patient data were completely anonymized and processed in encrypted form on a secure computer. The data of the study enrolment defined an index date for the analyses of problems identified by routine screening as compared to advanced screening. 

**Figure 1:**
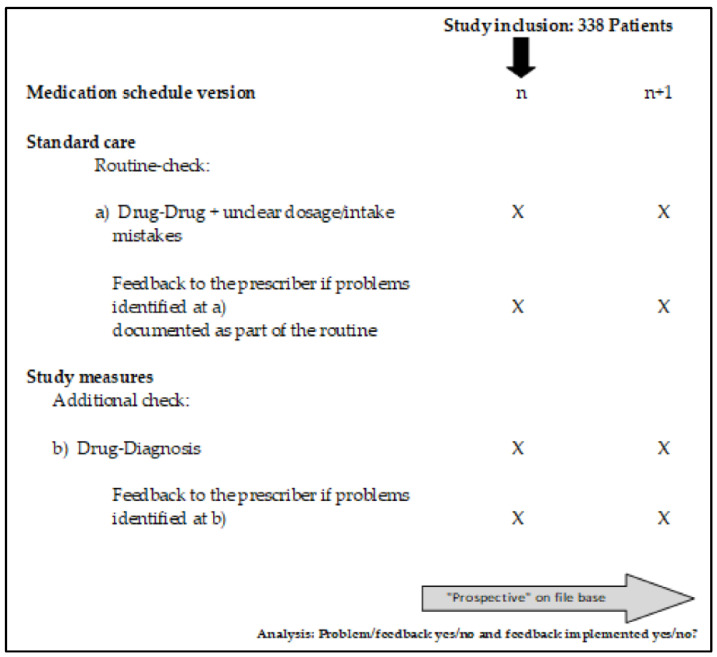
Overview of standard care and study measures.

The index prescription (*n*) is the prescription at which the potential medication problem appears for the first time, and (*n* + 1) the prescription immediately following it, for which the identified and reported interaction can be corrected by the prescribing doctor (Figure 1). From this perspective, it can be seen which warnings reported by the pharmacy were resolved by the prescribing doctor and which remained unresolved despite the warning.

### 2.6. Study Objectives

The key study objectives were:

1. To assess and compare the proportion and type of drug–drug interactions recorded during medication checks in routine care, to the proportion and type of drug–diagnosis contraindications that can only be detected by the advanced medication review under study conditions with available additional data and personnel expenditure.
2. To assess the implementation of warnings by the prescribing physicians.

### 2.7. Statistical Analysis

In the descriptive analysis, the frequency of changes in medication by the prescribing physician after the pharmacy had reported a drug–drug interaction as well as drug–diagnosis contraindications was recorded.

All statistical descriptive analyses were performed using Microsoft^®^ Excel 2018 and Microsoft^®^ Access 2018 and SPSS 28 (IBM).

## 3. Results

### 3.1. Sample

**Figure 2:**
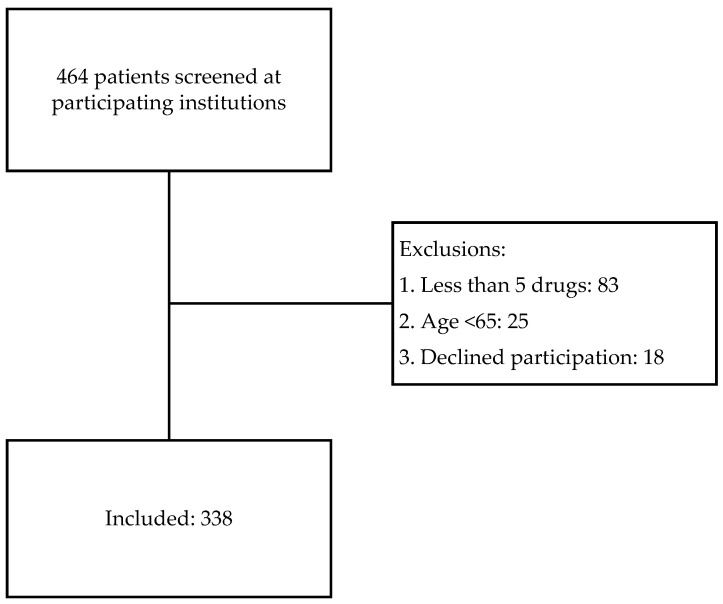
Flow-chart of screening, inclusion and exclusion of participants.

In total, 338 of 464 residents screened were included (Figure 2). 

The 338 participating patients had a mean age of 81.4 (±7.9, range 65–99) years, 241 (71.3%) were female. The mean number of medications taken was 7.8 (±2.4, range 5–14), the mean number of documented comorbidities was 5.5 (±1.9, range 3–11).

### 3.2. Identification and Communication of MRP by Routine and Advanced Medication Review

Key results are summarized in Table 1.

### 3.3. MRP Related to Documentation Problems

By routine review, a total of 43 documentation-related problems (i.e., mostly unclear or incomplete prescriptions) were identified (Table 2). 

### 3.4. MRP Related to Dosing, Double Medication, and Drug–Drug Interactions

In addition, 71 potential drug–drug interactions were flagged by the software. Of these, 14 (19.7%) were excluded from reporting to the physician due to lack of clinical relevance in the specific patients (Table 2 and Table 3). The most common drug–drug interactions were of the pharmacodynamics type (Table 2). The drugs most commonly involved in drug–drug interactions were antipsychotics (risperidone, melperone) with 35%, followed by antidepressants (citalopram, escitalopram, quetiapine, mirtazapine) with 28%, diuretics (furosemide, torasemide, hydrochlorothiazide) with 23%, and ACE inhibitors (ramipril) with 7%. The remaining 7% were distributed among other drugs.

#### MRP Related to Diagnoses

Of 69 prescriptions flagged as potentially contraindicated given the patient’s underlying diagnoses, 21 (30.4%) were excluded from reporting to the prescribing physicians due to a lack of clinical relevance (mostly related to outdated or imprecise information in the official SMPC not reflecting the current standard of care). Of the remaining 48 drug–diagnosis contraindications found, 12 were only reported as “potentially” contraindicated, since a final assessment could not be made due to the lack of more detailed clinical data (for example for a drug contraindicated in chronic heart failure “NYHA II” when only “chronic heart failure” was documented). The main diagnoses and related drugs involved are shown in Table 4.

### 3.5. Consequences of Alerts Generated by Routine and Advanced Medication Review

All 43 rather documentation-related problems detected by routine review were resolved by the pharmacy after a one-off consultation with the prescribing physician.

Of the 57 specific drug–drug interactions detected and reported by routine review, 35 (61.4%) were eventually resolved, 26 after the first report to the doctor, and 9 after a second report to the doctor (Table 2 and Table 3).

For 80.6% of the alerts that did not lead to a change of therapy, the physicians provided feedback for why the prescription was not changed after the alert. The explanations given as feedback from the doctors for not changing a medication flagged as (potential) MRP were lack of alternative therapy (61%), lack of clinical relevance (19%), and 20% remained unchanged and without feedback after the third alert. 

The majority (78.0%) of MRP related to documentation problems, drug interactions and unspecific problems with taking medication could be resolved by communication between the pharmacy and the prescribing physician. In contrast, only 51.2% of the alerts related to potential diagnosis-related contraindications were resolved, with lack of alternative therapy and lack of clinical relevance as the physician´s main explanations for not changing the medication related to an alert. 

## 4. Discussion

Our study involving 338 geriatric patients treated by 32 physicians revealed the following major findings: 

Of 148 potential MRP reported to physicians, 100 (68%) were related to documentation problems and drug–drug interactions typically detected by German pharmacy routine, while 48 (32%) reported that MRP were related to underlying diagnoses and could only be detected by advanced review. 

Of the alerts generated with the support of medication software or based on the official SMPC, 14 (19.7%) of the drug–drug and 21 (30.4%) of the drug–disease-related contraindications were deemed technically correct, but clinically inadequate and not communicated to the physician. 

The physicians followed 100% of the recommendations made in case of documentation-related alerts as compared to 61.4% of the routine alerts (reported drug–drug interactions) and 52.1% of the advanced alerts. 

Documentation errors and drug–drug interactions constituted the major proportion of MRPs identified by the pharmacist’s review. The implementation rate of 61.4% for reported drug–drug interactions correlates with data of previous studies [14].

The drug–drug interactions observed were most frequently associated with a risk for prolongation of the QT time, electrolyte disorders, or impairment of consciousness. With the exception of a lower rate of DDI related to bleeding risks (as compared to the literature [15]), this is largely in line with the results of other studies [16,17]. There is a good correlation between the drugs identified in the present study as involved in interactions, and drugs that are often prescribed in geriatrics (antipsychotics, antidepressants, diuretics, ACE inhibitors) [18,19].

### 4.1. Drug Diagnosis Contraindications

Furthermore, in line with previous studies [20], a relevant proportion of potentially contraindicated prescriptions was found to be related to clinical diagnoses.

In the present study, there was a good overall correlation between detected drug-disease interactions and diseases that occur frequently in old age [21,22], which confirmed the relevance of detecting drug–diagnosis contraindications in a geriatric population. An amount of 37.7% of all detected and 32.4% of all reported MRP were identified only after consideration of clinical diagnoses. This is a relevant proportion, but still less than one would expect based on the literature. In an analysis of ADE and medication errors in patients presented at an emergency department, originating from the same region as the present study, only 10% could be identified based on medication data alone (i.e., the data typically available to a pharmacist), the rest required consideration of additional clinical data [8]. This may constitute a somewhat unfair comparison though, because in emergency patients, the diagnoses may play a more critical role with respect to the sensitivity to adverse drug effects, as the patients may be even more critically ill than the average residents of a nursing home. Moreover, in the previous study, the complete patient records were available and all cases were reviewed by an interdisciplinary team involving pharmacists, emergency physicians, and clinical pharmacologists. 

In many clinical settings, medication reviews by pharmacists are rather “substance-oriented”, with a focus on drug–drug interactions and only limited consideration diagnoses. This may be explained by the more substance-oriented focus of the pharmacist’s training, and by the fact that often only the patient’s prescription or medication list is available to them. Therefore, an in-depth consideration of diagnoses in medication reviews in routine care cannot be expected from pharmacists in many clinical settings. Still, the present data indicate that a fairly basic sharing of key clinical diagnoses and the implementation of an “off-the-shelf” medication software can improve consideration of at least some key diagnoses for medication safety. Technically, it is already possible today to make diagnostic and laboratory data from the doctor or nursing home available to the local pharmacy. However, the fragmented structure of the German health care system in combination with a very strict interpretation of the European data protection laws constitute significant obstacles to any sharing or exchange of electronic health records between the different health care providers of a patient. 

Making some key data available across the board would not only benefit senior citizens living in retirement and nursing homes, but also other patients whose medication data can be routinely accessed by the patient’s pharmacists. A broad implementation would benefit from a framework of a truly digital nationwide medication plan, coupled with remuneration for additional work performed by the pharmacists and physicians.

Formally, no additional postgraduate training is required for this in Germany, but the broader consideration of clinical diagnoses should be matched by the appropriate postgraduate training for pharmacists. A recent report indicates that, so far, approximately 20% of the working German pharmacists already have participated in curricula for specialization as a “specialist pharmacist” (Fachapotheker) or sub-specializations, which specifically address the detection and prevention of medication related problems [23]. These include General Pharmacy (Allgmeinpharmazie), Clinical Pharmacy (Klinische Pharmazie), Medication Information (Arzneimittelinformation), Geriatric Pharmacy, and Medication Management in Hospitals (Medikationsmanagement im Krankenhaus).

### 4.2. Communication and Solution of Potential MRP 

In line with previous studies, a systematic medication review enables a large number of interactions to be recognized by the pharmacy. Pharmacist-led interventions have the potential to reduce the incidence of MRP in older people living in nursing homes [24,25]. The observed overall rate of 78.0% of MRPs detected via routine review and resolved in response to the pharmacist’s alerts correlates well with data reported by Bitter et al. in 2019 and Lexow et al. in 2022 for geriatric residents in German long-term care facilities [26,27].

The change in therapy due to reported drug–diagnosis contraindications in 52.1% of the cases showed that the standard availability of diagnoses in the pharmacy as well as suitable software solutions could already make a relevant contribution to improve drug therapy safety. The lower rate of resolved potential diagnosis-related MRP, as compared to drug–drug interaction-related MRP, is not surprising and most likely can be attributed to the more complex clinical judgements required and the still limited diagnosis data available to pharmacists. What looks like a prescribing error to a pharmacist, who has only a set of abbreviated diagnoses available, may still be considered acceptable or even clinically indicated by a physician to whom the complete clinical record is available and who also knows the personal preferences of the patient. 

Which diagnoses-related MRP can then be properly assessed in the routine by the pharmacist, and which cannot, still requires further research. In this respect, it has to be considered that, up to now, the interpretation of medical diagnoses is not in the primary focus of the rather substance-oriented training of pharmacists in many countries such as Germany. Theoretically, diagnosis-related contraindications can be called up in the pharmacy from the respective medicinal product information, but to what extent these have practical relevance on the medical side or not, must be investigated in individual cases. 

Finally, it needs to be emphasized that alerts elicit and increase the need for communication between pharmacists and the prescribing physicians. This sometimes also requires the different physicians of a patient to better communicate and coordinate their therapies as well. Critical findings may even create a compelling legal obligation (prevention of harm) for their timely communication and with it, issues of responsibilities. How, with how much effort, and how fast to contact the physician, or the patient, and who is finally responsible that the communication works? Considering the possibility of false alerts and the possible misdirection of valuable clinical resources to address them, a balance has to be found between the need and legal obligation to minimize harm to the patients, and the effort and resources available and dedicated to generate and follow-up on alerts in the clinical routine.

In the present setting that involved patients participating in a program providing a common platform that can be used by physicians and pharmacists identifying, communicating, and resolving MRP may have been easier than in a setting where physicians and pharmacists practically have no direct means of communication or, worse, no knowledge of each other. 

In this respect, communication channels that involve the patient (pharmacist-doctor-patient) remain unclear, as these have not yet been specifically regulated, at least not in Germany.

In the present setting of care, the pharmacy contacts the prescribing physician via fax in order to induce and receive a decision regarding a possible change of therapy. The responsibility for the medical treatment of patients lies solely with the physician and therefore the pharmacy only gives advice to the prescribing doctor without contacting the concerned patient. The fact that 22.0% of alerts to the doctor remained unresolved shows that the collaboration between doctors and pharmacists regarding the management of drug interactions among residents of retirement and nursing homes can still be optimized. 

### 4.3. Strengths and Limitations

This study only made use of the data available at the care facility in the routine care setting of geriatric patients in Germany, which can be considered a limitation as well as a strength. A limitation, because not all possible medication-related problems could be detected and addressed based on the rather limited routine documentation available in German ambulatory care, and a strength, because the study reflects a detection and communication of medication-related problems that is representative as well as technically possible and compatible with the resources of routine care. The study was single-centric with respect to the pharmacy involved, but multi-centric with respect to the physicians and geriatric care centers involved. The patients in this study can be considered fairly representative of the large population of the German elderly living in a nursing home. Given the universal relevance of diagnosis data in medication safety, the present observations can most likely be generalized to settings where several pharmacists and physicians independently care for the same patient. It has already been shown in the setting of an emergency department that consideration of diagnoses is required for the detection of a large proportion of medication-related problems [8]. Therefore, the impact of an improved availability and consideration of diagnoses by pharmacists should be investigated in other settings and populations such as in ambulatory patients.

The rate of MRP detected does not represent the possibly higher rates of MRP detected at the very first medication reviews because in the present setting, many problems may have been detected by previous medication reviews. Therefore, documentation problems, or drug–drug interaction-related problems were more likely to be related to the new prescription that triggered the review, as compared to diagnosis-related MRP which were not systematically assessed for any of the drugs on the medication plan in previous medication reviews.

## 5. Conclusions

Extended consideration of clinical diagnoses in medication reviews performed by pharmacists can improve patient safety by measurably increasing the number of identified and resolved MRPs. However, even the consideration of only key diagnoses already requires an extended supporting infrastructure for sharing clinical data between a patient’s physician(s) and pharmacist(s) that exceeds what is currently available in many places. Furthermore, it also requires an appropriate—clinically focused—training of pharmacists and an agreed upon process involving all stakeholders for communicating and resolving MRPs. While efforts in pharmacist training are already increasing, supporting infrastructure and communication processes remain to be established in many routine settings, with lack of incentives and resources (i.e., reimbursement of efforts) being the most likely obstacles, at least in Germany.

## Figures and Tables

**Table 1:** Prospective assessment of routine and advanced review.

	Index Prescription Triggering Medication Review	First New Prescription after Index Prescription Triggering the Follow-Up Medication Review
Medication reviews	338 (100%)	338 (100%)
Potential MRP detected by Routine review (% of cases with medication review)	114 (33.7%)	45 (13.3%)
Medication alerts related to MRP detected by Routine review (% of potential MRP)	100 (87.7%)	26 (57.8%)
Alert resolved at follow-up medication review (% of medication alerts at index prescription)	78 (78.0%)	Not applicable
Potential MRP detected by advanced review (% of cases with medication review)	69 (20.4%)	44 (13.0%)
Medication alerts related to MRP detected by advanced review (% of potential MRP detected by advanced review)	48 (69.6%)	34 (77.3%)
Alert resolved at follow-up medication review (% of medication alerts for advanced review at index prescription)	25 (52.1%)	Not applicable
All potential MRP detected (% of cases with medication review)	183 (54.1)	89 (26.3%)
Medication alerts related to MRP (% of all potential MRP)	148 (80.9%)	60 (67.4%)
Alert resolved at follow-up medication review (% of all medication alerts)	103 (69.6%)	Not applicable

**Table 2:** Drug–Drug Interaction related MRP reported and resolved.

Drug–Drug Interactions	Problems Reported to Physicians	Solved after Report
Reported interactions	57	35
QT-Time prolongation	31	19
Electrolyte disorders	10	8
Impairment of consciousness	9	6
Others	7	2

**Table 3:** MRP reported to physicians (MRP alerts) and resolved.

	Problems Reported to Physicians	Solved after Report *n* (%)
**All**	**148**	**103 (69.6)**
**Documentation-related MRP**	**43**	**43 (100)**
Missing or wrong dosing schedule	32	32 (100)
Unclear/missing dosage	10	10 (100)
Unclear treatment termination or pause	1	1 (100)
**Substance-related problems (Contraindicated or critical drug–drug interactions)**	**57**	**35 (61.4)**
**Diagnosis-related MRP**	**48**	**25 (52.1)**

**Table 4:** Diagnosis-related MRP observed.

Diagnoses	Contraindicated Drugs
Renal failure (34%)	Citalopram/Metformin
Morbus Parkinson (33%)	Pipamperon/Sulpirid
Convulsions (25%)	Citalopram/Sulpirid
Others (8%)	Others

## Data Availability

Not applicable.
